# Everybody's Page

**Published:** 1890-09-06

**Authors:** 


### EVERYBODY'S PAGE.

SCARLET fever is sometimes so mild that it is only diagnosed by the nephritis which follows.

THE doctors of Pearce's Invalid Hotel in the States, employ eight phonographs into which they speak their prescriptions, &c., which are afterwards type-written by two young ladies and then sent to the dispensary.

DOCTOR BROUARDEL has read to the Paris Academy of Medicine a paper by Doctor Paul Garnier on the effects of the alcohol sold in Paris upon the nervous systems of those who consume it. Since 1877 the number of madmen has increased three-fold upon that of the preceding thirteen years.

ONE of the many beautiful legends about St. Theresa is that she arrived at Toledo to found a convent with only four ducats. She was greeted with ridicule, but merely said, "Theresa and four ducats can do nothing; but God, Theresa, and four ducats can do anything." The convent was founded. In the garden of the convent at Avila, where Theresa is buried, is an apple tree, planted by her hands, the fruit of which is believed to cure every species of female disorder.

AN interesting article in the *Westminster Review* gives a description of the handsome crematorium at Milan and its manner of working. Two systems of cremation are followed at Milan, by one of which the body is burned in a furnace surrounded by wood and charcoal, while by the other the combustion is brought about through a number of jets of gas which cast their heat upon the furnace from all sides. When wood and charcoal are employed, about six hundred pounds of wood and one of charcoal are found necessary, and the process lasts two hours. When gas is used, all that is consumable in the body is burned up in less than 50 minutes. The body may, in ordinary cases, be introduced into the furnace with or without the coffin. But if death has been caused by some infectious disease the coffin and body must be burned together. The weight of the remains after cremation, in the form of bones and dust, is about four pounds. They are in colour pure white, tinged here and there with a delicate pink; and it is a rule never to touch them with the hand. The bones and vestiges of bones (which are for the most part burnt into powder) are taken up with silver tongs, while the ashes are removed from the furnace with a silver shovel, to be placed on a silver dish and then deposited in an urn for retention in the cinerarium. Here the ashes are preserved in separate compartments, each with a suitable inscription beneath it. The cost of cremation is 25 francs to a member of the society for extending cremation in Italy, or 50 francs in the case of non-members.

NEURALGIA is sometimes treated in New York by refrigeration, produced by a spray.

DR. DANA says that a sound nervous system cannot be seriously disordered by local irritations, and that undue attention is paid to reflex nervous disorders of the nose and pharynx.

A SIMPLE test for the purity of air in a room is to put a saucer of clear lime-water on the floor near the bed, and let it remain all night. In the morning if the air is foul, the saucer will be found covered with a thin white crust of carbonate of lime.

A SERIOUS and irksome task devolving upon the directors of charitable societies is the collection of funds. When benevolent people who appreciate and wish to aid these societies learn to give in response to printed or written requests, instead of waiting for personal solicitation, they will increase their gifts without added cost to themselves, by the great saving in the cost of collection. It is an astonishing thing that in these days of common-sense it is necessary for hospitals to employ a gentlemanly young man to go round and "beg" for subscriptions.

A PROOF of the paragraph we gave here last week on the control of children is the following story from last Friday's daily papers: James Godfrey, living at Westmoreland-place, was charged at Worship-street with ill-treating his son George, aged seven years. Mr. Morris prosecuted for the National Society for the Prevention of Cruelty to Children. William Lewis, an elderly man, said that on August 7th he saw the boy in the street. There were marks as of a strap on the boy's back from the shoulders downwards to the hip. The skin was knocked off one shoulder and one elbow. Police-constable 461 G said he saw the boy, and examined him at the station. The marks of bleeding on the boy were "two patches about the size of a ha'penny." Police-constable 410 G said that he went with the boy to the station, and examined him, and found his shirt "saturated with blood." Cross-examined, the constable said he had not heard the other constable say there were two marks about the size of a halfpenny; he thought the boy's shirt was "saturated." There were marks of a buckle on the boy's back. A letter from a Board school stated that the boy was a truant, untruthful, and dishonest. A letter giving defendant a good character was also read. The father was called, and said that he chastised the boy because he had thrown stones and broken a window. Mr. Bushby said the evidence as to the use of the buckle was too vague. The evidence did not sustain the charge. The summons was dismissed, and an order made against the society for £1 1s. costs.

